# Young athletes return too early to knee-strenuous sport, without acceptable knee function after anterior cruciate ligament reconstruction

**DOI:** 10.1007/s00167-017-4747-8

**Published:** 2017-10-14

**Authors:** Susanne Beischer, Eric Hamrin Senorski, Christoffer Thomeé, Kristian Samuelsson, Roland Thomeé

**Affiliations:** 1 Unit of Physiotherapy, Department of Health and Rehabilitation, Institute of Neuroscience and Physiology, Sahlgrenska Academy, University of Gothenburg, Box 455, 405 30 Gothenburg, Sweden; 2 Sportrehab Sports Medicine Clinic, Stampgatan 14, 411 01 Gothenburg, Sweden; 3 Department of Orthopaedics, Sahlgrenska University Hospital, Mölndal, Sweden; 4 Department of Orthopaedics, Institute of Clinical Sciences, The Sahlgrenska Academy, University of Gothenburg, Gothenburg, Sweden

**Keywords:** Adolescents, Knee, Rehabilitation, Muscle function, Return to sport, Register study

## Abstract

**Purpose:**

The purpose of this study was to evaluate the return to knee-strenuous sport rate, muscle function and subjective knee function among adolescent patients (15–20 years of age) and adult patients (21–30 years of age) 8 and 12 months, respectively, after anterior cruciate ligament (ACL) reconstruction. It was hypothesised that no differences in outcome would be found between age groups at 8 or 12 months after ACL reconstruction.

**Methods:**

Cross-sectional data from five tests of muscle function, from the Knee injury and Osteoarthritis Outcome Score (KOOS) and the Tegner Activity Scale (Tegner), performed at 8 and 12 months after a primary ACL reconstruction, were extracted from a rehabilitation outcome register. A total of 270 (51% women) athletes, aged 15–30 years, who were all involved in knee-strenuous sport prior the injury, were included at 8 months after ACL reconstruction. At 12 months 203 (51% women) were included. The return to knee-strenuous-sport rates and the rate of achieving a limb symmetry index of ≥ 90% in all five tests of muscle function, defined as recovery of muscle function, and subjective knee function scores, as measured with the KOOS, were compared between age groups.

**Results:**

The adolescent patients had a higher (50%) return to knee-strenuous sport rate compared with the adult patients (38%) 8 months after ACL reconstruction (*p* = 0.04). At the 12-month follow-up, no difference was found between the age groups; 74 and 63%, respectively. At the 8-month follow-up, 29% of the patients, in both age groups, who had returned to sport had recovered their muscle function in all five tests of muscle function. At the 12-month follow-up, the corresponding results were 20% for the adolescents and 28% for the adult patients. No difference in mean KOOS scores was found between the age groups at 8 or at 12 months after ACL reconstruction.

**Conclusion:**

The majority of young athletes make an early return to knee-strenuous sport after a primary ACL reconstruction, without recovering their muscle function. To set realistic expectations, clinicians are recommended to ensure that young athletes receive information about not to return before muscle function is recovered and that this may take longer time than 12 months.

**Level of evidence:**

II.

## Introduction

One of the most devastating consequences when returning to sport (RTS) after anterior cruciate ligament (ACL) reconstruction is a subsequent ACL injury. Adolescent patients (15–20 years) run a remarkably increased risk of a second ACL injury; up to 30% will require a new ACL reconstruction within the first two years after RTS [1, 2, 13, 23, 27, 34, 36]. Two identified risk factors for an additional ACL reconstruction are primary ACL reconstruction at younger age, i.e., age < 20 years [9, 19, 20, 28], and higher activity level [2, 9, 19, 28, 36].

Recent studies have highlighted and discussed the importance of delaying RTS to at least 9 months to lower the risk of a subsequent ACL injury [14, 23]. Further, restoring muscle function before RTS is regarded as another important factor [14, 21] in lowering the re-injury risk. The limb symmetry index (LSI) is the most frequently reported criterion for assessing whether strength and hop performance is classified as normal or abnormal. An LSI of > 90% is commonly regarded as sufficient for both leg muscle strength and hop performance after ACL injury and reconstruction [4, 22, 31]. However, several studies report that many patients do not achieve this level in a combined battery of strength and hop tests 6–12 months after ACL reconstruction [11, 14, 17, 32, 35]. Younger age appears to favour returning to the pre-injury level of sport [3]. However, it has not previously been reported whether younger athletes (15–20 years old) recover their muscle function before they return to knee-strenuous sport.

Patients have been reported to have high expectations on the overall condition of the knee joint 12 months after an ACL reconstruction, especially younger patients, patients without previous knee surgery, and highly active patients [10]. Furthermore, patients who return to sport have been found to have less impairment during sport and recreation and enhanced knee-related quality of life after ACL reconstruction as compared to patients who do not return to sport [8, 16]. However, it is not known whether there is any discrepancy in subjective knee function between patients of different ages who return to sporting activities.

Taken together, patients under the age of 20 who are involved in knee-strenuous sport at the index ACL injury constitute a high-risk group in terms of sustaining a subsequent ACL injury. The timing of RTS and recovery of muscle function have been reported to be important aspects to consider in the RTS decision. To our knowledge, no previous study has evaluated these aspects specifically in adolescent patients. The purpose of this study was, therefore, to evaluate the return to knee-strenuous-sport rates, muscle function and subjective knee function among adolescent patients (15–20 years) and adult patients (21–30 years) 8 and 12 months, respectively, after ACL reconstruction. It was hypothesised that no differences in outcome would be found between the age groups at 8 or 12 months after ACL reconstruction.

### Materials and methods

**Fig. 1:**
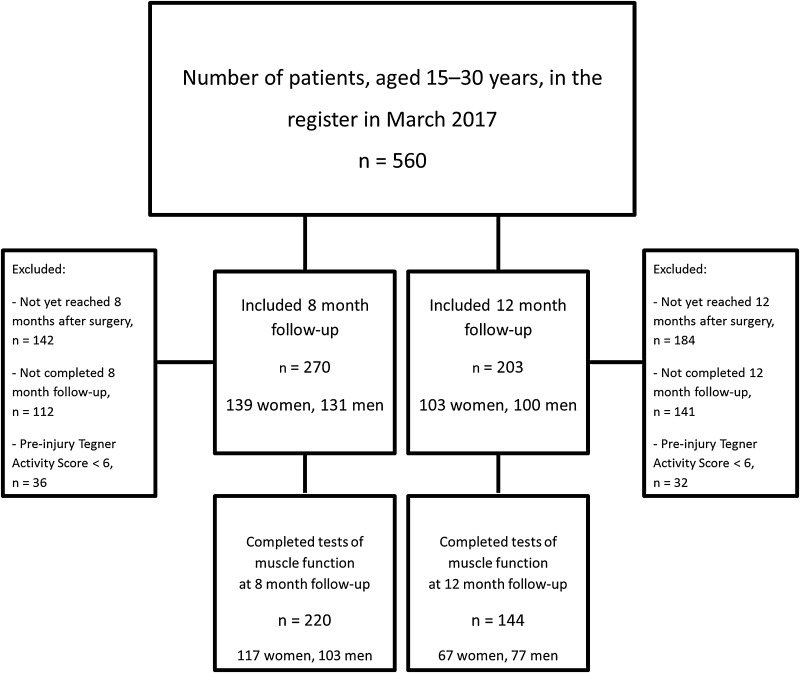
Flow chart of inclusion and exclusion criteria

The study was performed as a prospective, observational register study based on data from a rehabilitation outcome register. The register is located in the western part of Sweden and was established in September 2014. At the end of March 2017, the register comprised more than 1,200 patients, of which 560 patients had undergone a unilateral ACL reconstruction and were between 15 and 30 years of age at the time of surgery (Fig. 1). The register consists of two parts: a battery of validated patient-reported outcome measurements (PROMs) and a battery of muscle function tests for leg-muscle strength and hop performance. Patients were regularly assessed according to a predefined schedule of follow-ups at 10 weeks, 4, 8, 12, 18 and 24 months and then every 5 years after their index ACL injury or reconstructive surgery. All patients were given written information about the study. Informed consent was obtained and the rights of subjects were protected.

### Participants

Cross-sectional data from the 8- and 12-month follow-ups were extracted from the rehabilitation outcome register. Patients with a unilateral ACL injury who had undergone ACL reconstruction between September 2013 and July 2016 were eligible for inclusion. A further inclusion criterion was a pre-injury self-reported physical activity level on the Tegner Activity Scale (Tegner) [30] of ≥ 6, i.e., involvement in a knee-strenuous sport. A flow chart of the inclusion and exclusion criteria is presented in Fig. 1.

### Procedure

#### Muscle function

Assessments of muscle strength were performed at the House of Sport Science, University of Gothenburg, by educated test administrators, all registered physiotherapists. Two methods of muscle strength assessment were used in this study. Initially, isometric tests were performed using a David F200 DMS-EVE (David Health Solutions Ltd, 2013, Finland) and these values contribute to about 30% of the total muscle strength data. The isometric test evaluated peak torque in knee extension at 60° of knee flexion and knee flexion at 30° of flexion. In December 2015, these tests were replaced by an isokinetic concentric strength test of knee extension and knee flexion using a Biodex System 4 (Biodex Medical Systems, Shirley, New York) [33]. Both versions of the strength test were performed according to a structured protocol, consisting of a 10-min warm-up on an exercise bike, followed by a warm-up procedure and familiarisation with sub-maximum practice trials. The isometric tests were performed unilaterally and the patients were instructed to extend/flex their knee with maximum effort for 2–4 s. The isokinetic tests were performed at an angular velocity of 90°/s after a warm-up protocol similar to that for the isometric tests. After warming up, 3–5 maximum isometric/isokinetic trials were performed with 40 s’ rest in between. For both tests, the best trial for extension and flexion torque for each leg was documented and used for further analysis.

After strength testing, three hop tests were performed in the following order: unilateral vertical hop; unilateral hop for distance; and unilateral side hop. High test–retest reliability for the three different tests in the battery of hop tests has been reported [15]. For the vertical hop and the hop for distance, the patients in the present study performed three to five practice trials, followed by three maximum trials. However, if the subject or the test administrator felt that an even better result could be achieved, one to two additional hops were allowed. The side hop was tested once and started with ten practice hops, followed by as many hops as possible for 30 s over two lines, 40 cm apart. Three minutes of rest were given between legs for the side-hop test [15]. The best trial for each leg in each test was used for further analysis.

#### Patient-reported outcome measurements

To assess the pre-injury and the present level of physical activity, the Tegner was used. The Tegner is graded from 1 to 10, with 1 representing the least strenuous knee activity and 10 representing the most strenuous knee activity, such as rugby and international soccer. The Tegner has been reported to have acceptable test–retest reliability (ICC = 0.8) [6]. In the present study, return to sport was defined as returning to a Tegner of 6 or higher, i.e., a knee-strenuous sport [16].

The Knee injury and Osteoarthritis Outcome Score (KOOS) [25] was used to assess patients’ opinions about their knee and associated problems. In the present study, the subscales of pain, other symptoms, function in sport and recreation and knee-related quality of life were used. The KOOS has been reported to have acceptable test–retest reliability with an ICC ranging from 0.85 to 0.93 for the subscales used in the present study [25].

Ethics Approval has been obtained from the Regional Ethical Review Board in Gothenburg, Sweden (registration numbers: 265-13, T023-17).

### Statistics analysis

Statistical analysis was performed using the statistical package for the social sciences, SPSS (version 22, 2013; SPSS Inc., Chicago, IL, USA). The results of muscle function tests were presented as the LSI. The LSI was used to analyse results from the muscle function tests. The LSI was defined as the ratio of the injured side and the non-injured side expressed as a percentage. In this study, recovery of muscle function was defined as achieving an LSI of ≥ 90% in all tests of muscle function.

Descriptive statistics for patient demographics and outcomes were reported as the mean and standard deviation (SD) for parametric data and as the median, interquartile range and minimum and maximum for non-parametric data. For between-group comparisons, the Mann–Whitney *U* test and an independent *t* test were used for non-parametric and parametric data respectively. The Chi-square test was used to analyse associations between categorical variables.

A power analysis based on a previous study [32] suggested that 36 persons/group were needed for a power of 80% to detect a significant difference in knee extension strength, presented as LSI, between groups with a variance of 10% point and a significance level of 5%.

## Results

A total of 270 (51% women) patients met the inclusion criteria at 8 months. At 12 months, 203 (51% women) patients were included. In all, 42% (114/270) and 67% (136/203) of the cohort reported that they had returned to knee-strenuous sport 8 and 12 months, respectively, after ACL reconstruction (Tables 1, 2).

**Table 1:** Patients’ demographics for patients at 8 months after anterior cruciate ligament reconstruction

Demographics	Returned to knee-strenuous sport [Tegner Activity Score ≥ 6) (*n* = 114)]		*p* value	All included patients (*n* = 270)		*p* value
	Age 15–20 years (*n* = 51)	Age 21–30 years (*n* = 63)		Age 15–20 years (*n* = 102)	Age 21–30 years (*n* = 168)	
Age at reconstruction (years)						
Mean ± SD	17.2 ± 1.2	24.5 ± 2.7	n.a	17.4 ± 1.3	24.9 ± 2.6	n.a
Length (cm)						
Mean ± SD	172 ± 10	179 ± 8	0.010^a^	173 ± 10	177 ± 9	0.008^a^
Weight (kg)						
Mean ± SD	72 ± 11	79 ± 13^b^	0.002	70 ± 11	76 ± 13^b^	0.001^a^
Phys activity level						
Pre-injury^c^						
Mean ± SD	9 ± 1	9 ± 1	n.s.^d^	9 ± 1	8 ± 1	0.001^d^
Median [min–max]	9 [7–10]	9 [6–10]		9 [6–10]	8 [6–10]	
ICR	8–10	8–9		8–9.25	7–9	
Gender, *n* (%)						
Women	30 (59)	19 (30)	0.002^e^	69 (67)	70 (42)	< 0.001^e^
Men	21 (41)	44 (70)		34 (33)	98 (58)	

*p* value for comparison between the age groups,* p* ≤ 0.05 indicate statistical significance

*n.a*. not applicable, *n.s*. not significant

^a^Independent *t* test; ^b^missing data for one patient; ^c^as measured with Tegner Activity Score, score range 0–10; ^d^Mann–Whitney *U* test; ^e^Chi-square test

**Table 2:** Demographic data stratified by age groups in patients who had returned to knee-strenuous sport and for the total cohort 12 months after anterior cruciate ligament reconstruction

Demographics	Returned to knee-strenuous sport [Tegner Activity Score ≥ 6) (*n* = 136)]		*p* value	All included patients (*n* = 203)		*p* value
	Age 15–20 years (*n* = 56)	Age 21–30 years (*n* = 80)		Age 15–20 years (*n* = 76)	Age 21–30 years (*n* = 127)	
Age at reconstruction (years)						
Mean ± SD	17.2 ± 1.2	24.3 ± 2.6	n.a	17.3 ± 1.2	24.8 ± 2.8	n.a
Length (cm)						
Mean ± SD	171 ± 25	178 ± 9	0.002^a^	170 ± 22	177 ± 9	0.004^a^
Weight (kg)						
Mean ± SD	71 ± 10^b^	77 ± 11	0.002^a^	71 ± 11^c^	75 ± 11	0.005^a^
Phys activity level						
Pre-injury^d^						
Mean ± SD	9 ± 1	9 ± 1	n.s.^e^	9 ± 1	8 ± 1	0.001^e^
Median [min–max]	9 [7–10]	9 [6–10]		9 [7–10]	8 [6–10]	
ICR	8–9	8–9		8–9	7–9	
Gender,* n* (%)						
Women	65.5 (38)	27 (34)	< 0.001^f^	69 (54)	40 (51)	< 0.001^f^
Men	35.5 (20)	53 (66)		31 (24)	60 (77)	

*p* value for comparison between the age groups,* p* ≤ 0.05 indicate statistical significance

*n.a*. not applicable, *n.s*. not significant

^a^Independent* t* test; ^b^missing value for one patient; ^c^missing value for two patients; ^d^As measured with Tegner Activity Score, score range 0–10; ^e^Mann–Whitney *U* test; ^f^Chi-square test

**Table 3:** Results from tests of muscle function, including proportion (%) of patients achieving normal Limb Symmetry Index (LSI) (≥ 90%) at 8 months

LSI mean ± SD (%) proportion (%)	Returned to knee-strenuous sport (Tegner Activity Score ≥ 6) (*n* = 95)		*p* value
	Age 15–20 years (*n* = 45)	Age 21–30 years (*n* = 50)	
Quadriceps	94 ± 9	94 ± 12	n.s
	80	72	
Hamstrings	100 ± 10	100 ± 14	n.s
	84	80	
Vertical hop	88 ± 10^a^	88 ± 15^b^	n.s
	46	54	
Hop for distance	96 ± 8^b^	95 ± 10^a^	n.s
	81	80	
Side hop	99 ± 15^c^	93 ± 15^d^	n.s
	78	72	

Statistical significant difference between groups at *p* value < 0.05 using independent *t* test

*n.s*. not significant

^a^Missing value for one patient; ^b^missing value for two patients; ^c^missing value for four patients; ^d^missing values for three patients

At 8 months after ACL reconstruction, there was a significant difference in the return to knee-strenuous sport rate between the adolescent patients and the adult patients, 50 and 38%, respectively (*p* = 0.044). No significant difference in the return to knee-strenuous- sport rate was found between females and males among the adolescent patients (44 and 64%, respectively). However, among the adult patients, males had a significantly higher rate of return to knee-strenuous sport compared with females (45 and 27%, respectively; *p* = 0.019) (Table 2).

No significant difference was found in the rate of return to knee-strenuous sport between the adolescent patients and the adult patients at 12 months after ACL reconstruction, 74 and 63%, respectively. Furthermore, no difference in the return to knee-strenuous sport rate was found between males and females among either the adolescent or the adult patients.

In all, 29% of both the adolescent (13/45) and the adult patients (14/49), who had returned to knee-strenuous sport, achieved an LSI of ≥ 90% in all five tests of muscle function at 8 months after ACL reconstruction. At the 12-month follow-up, 20% of the adolescent patients (8/40) and 28% of the adult patients (17/60) (n.s.) achieved an LSI of ≥ 90% in all five tests of muscle function. No difference in the proportion of patients who achieved an LSI of ≥ 90% in all five tests of muscle function or in the mean LSI was found between the adolescent and the adult patients or between females and males in any age group in any of the separate tests in the test battery at either 8 or 12 months after ACL reconstruction (Tables 3, 4).

**Table 4:** Results from tests of muscle function, including proportion (%) of patients achieving normal Limb Symmetry Index (LSI) (≥ 90%) at 12 months

LSI mean ± SD (%) proportion (%)	Returned to knee-strenuous sport (Tegner Activity Score ≥ 6) (*n* = 100)		*p* value
	Age 15–20 years (*n* = 40)	Age 21–30 years (*n* = 60)	
Quadriceps	98 ± 9	97 ± 11	n.s
	83	83	
Hamstrings	99 ± 10	99 ± 14	n.s
	80	78	
Vertical hop	92 ± 14^a^	90 ± 12^b^	n.s
	50	50	
Hop for distance	97 ± 7^a^	96 ± 7^b^	n.s
	78	83	
Side hop	101 ± 17^c^	97 ± 12^c^	n.s
	78	75	

Statistical significant difference between groups at *p* value < 0.05 using independent *t* test

*n.s*. not significant

^a^Missing value for four patients; ^b^missing value for two patients; ^c^missing value for three patients

**Table 5:** Mean (± SD) *Knee injury and Osteoarthritis Outcome Score* (KOOS) for patients who had returned to knee-strenuous sport stratified by age groups 8 months after anterior cruciate ligament reconstruction

Mean ± SD	Returned to knee-strenuous sport (Tegner Activity Scale ≥ 6 (*n* = 114)]		*p* value
	Age 15–20 years (*n* = 51)	Age 21–30 years (*n* = 63)	
KOOS			
Pain	89.9 ± 15.0	87.4 ± 10.2	n.s
Symptom	80.9 ± 16.5	76.3 ± 15.3	n.s
Sport	79.0 ± 19.3	74.1 ± 16.9	n.s
QoL	62.4 ± 21.0	62.4 ± 16.4	n.s

The possible score range for each subscale are 0–100; 0 = extreme knee-problem, 100 = no knee-problem

Statistical significant difference between groups at *p* value < 0.05 using independent* t* test

*n.s*. not significant; *Sport* function in sport and recreation, *QoL* knee-related quality of life

**Table 6:** Mean (± SD) *Knee injury and Osteoarthritis Outcome Score* (KOOS) for patients who had returned to knee-strenuous sport stratified by age groups 12 months after anterior cruciate ligament reconstruction

Mean ± SD	Returned to knee-strenuous sport (Tegner Activity Scale ≥ 6 (*n* = 136)]		*p* value
	Age 15–20 years (*n* = 56)	Age 21–30 years (*n* = 80)	
KOOS			
Pain	91.5 ± 9.6	89.5 ± 9.6	n.s
Symptom	84.1 ± 13.6	80.6 ± 15.0	n.s
Sport	83.8 ± 16.8	80.1 ± 15.7	n.s
QoL	69.7 ± 19.0	66.1 ± 16.5	n.s

The possible score range for each subscale are 0–100; 0 = extreme knee-problem, 100 = no knee-problem

Statistical significant difference between groups at *p* value < 0.05 using independent* t* test

*n.s*. not significant, *Sport* function in sport and recreation, *QoL* knee-related quality of life

No differences in any of the KOOS subscales were seen between the adolescent and the adult patients who reported that they had returned to knee-strenuous sport, at 8 or at 12 months after ACL reconstruction (Tables 5, 6). Furthermore, no differences in any of the KOOS subscales were found between females and males in either the adolescent or the adult patients.

## Discussion

The main finding in this prospective observational register study was that adolescent patients returned at a higher rate to knee-strenuous sport 8 months after ACL reconstruction compared with adult patients. Furthermore, fewer than 30% of all patients who had returned to knee-strenuous sport, at both follow-ups, achieved an LSI of ≥ 90% in all measurements in a battery of tests.

In the present study, there was a significant difference in the rate of return to knee-strenuous sport between adolescent and adult patients 8 months, but not 12 months, after ACL reconstruction. It has previously been reported that younger athletes are more likely to return to their pre-injury sport compared with their older counterparts 12 months after ACL reconstruction [3]. The difference in the result of the present study and the study by Ardern et al. [3] can be attributed to the fact that the present study only included young patients, aged 15–30 years, whereas Ardern et al. [3] based their systematic review on studies including patients up to 60 years of age. However, the present study indicates that the adolescent patients returned to knee-strenuous sport to a higher extent compared with the adult patients 8 months after the ACL reconstruction. It can be argued that the majority of the adolescent patients in the present study returned to knee-strenuous sport too early after ACL reconstruction. However, 8 months after ACL reconstruction, some patients are in a phase of rehabilitation where the transitioning to sport through sport-specific exercises, such as cutting and jumping, and gradual sports participation are commonly introduced or progress further in terms of intensity [4, 12, 29]. Some patients might even have begun to participate, at least in a modified manner, in knee-strenuous activities. There is a lack of evidence in the current literature with regard to adolescent patients in terms of the optimal progression of rehabilitation and the criteria patients need to meet before returning to unrestricted sports participation.

In the present study, only a minority, less than one-third, of both the adolescent and the adult patients achieved an LSI of ≥ 90% in all five tests of muscle function at 8 and 12 months after the ACL reconstruction. This is in line with previous studies reporting that 10–57% of patients achieve normal levels of muscle function [14, 32, 35] six to 12 months after ACL reconstruction. However, the apparent difference in the results of the present study and previous studies can be attributed to the younger population in the present study, including only patients who had returned to a knee-strenuous sport. Previous studies [11, 14, 17, 32, 35], as well as the present study, clearly demonstrate that the majority of the patients did not have recovered their muscle function once they returned to knee-strenuous sport after ACL reconstruction, in spite of the fact that the group’s mean LSIs are > 90% in all the individual tests in the battery. In a recently published study [35], the use of the LSI in RTS decisions has been questioned, as LSIs appear to overestimate knee function after ACL reconstruction and might not be sensitive enough to predict subsequent ACL injury. Furthermore, lower absolute levels of muscle function in the uninvolved leg might conceal an abnormal muscle function [18, 32]. Taken together, it appears that far too few patients have recovered their muscle function before returning to knee-strenuous sport. This is worrying and presents a major challenge that needs to be resolved. Considering the limitations of using the LSI, this problem can in fact be even worse. More effective rehabilitation and RTS criteria are suggestions of some areas that could be improved.

No difference in subjective knee function was found between the adolescent and the adult patients at either the 8- or the 12-month follow-up. No previous studies comparing adolescents and adults with respect to subjective knee function have been found. However, the results of the present study are in line with the results that have been suggested as a functional recovery among adult patients 12 months after ACL reconstruction, except for the KOOS subscale of knee-related quality of life [5, 24]. For this subscale, the patients in both age groups reported > 20-point lower scores at both the 8- and 12-month follow-up compared with scores that are considered to be equal to functional recovery. This could be an indication of a negative psychological response to the injury, the surgery, or the rehabilitation [7, 26]. However, knowledge of the psychological response in relation to return to sport among a young ACL population, aged < 20 years, is lacking.

Future studies may indicate whether the patients in the present study, who had returned to knee-strenuous sport as early as 8 months after ACL reconstruction, constitute a high-risk group for subsequent injuries. The high re-injury rate after ACL reconstruction among adolescent patients, as described in the literature [1, 2, 13, 23, 27, 34, 36], can be partly explained by the fact that the adolescent patients return to knee-strenuous sport too early, without achieving adequate muscle function.

This cross-sectional study has some limitations that were taken into account before conclusions were drawn. First, data relating to sport participation, as measured with Tegner, only reflect how knee strenuous the sports in which the patients participated actually were. Data relating to exposure, i.e., the frequency of participation, or whether the patients participated in modified or unrestricted training/competition, were not available. Second, two methods were used to assess the patients’ muscle strength. Both methods register peak torque at similar knee extension and flexion angles and in a similar seated position. Furthermore, the outcomes from the two methods are considered to be comparable, as they are presented as the LSI and not as absolute values. As a result, there is no reason to believe that this limits the opportunity to draw conclusions in the present study. Third, the adolescent patients comprised more women than men, as compared with the group of adults. However, a subgroup analysis of the adolescent and adult patients separately revealed no differences between genders in any of the outcome measurements. It was, therefore, assumed that the gender distribution in the present study did not limit the opportunity to draw conclusions. However, it is important to remember that the number of patients who performed the battery of tests was somewhat low when subgroups of ages were stratified by gender.

This is the first study to investigate differences in muscle function and subjective knee function between adolescents and adult patients who have undergone a primary ACL reconstruction. A large population of 270 and 203 young patients, respectively, was included at the two follow-ups. The patients were homogeneous in terms of age and pre-injury level of sport participation. Furthermore, the methods used for assessing strength and hop performance, as well the PROMs that were used, are all reliable and valid for evaluating patients after an ACL reconstruction.

## Conclusions

The majority of young athletes make an early return to knee-strenuous sport after a primary ACL reconstruction, without recovering their muscle function. To set realistic expectations, clinicians are recommended to ensure that young athletes receive information about not to return before muscle function is recovered and that this may take longer time than 12 months.
